# Pluripotent Stem Cell Therapies for Parkinson Disease: Present Challenges and Future Opportunities

**DOI:** 10.3389/fcell.2020.00729

**Published:** 2020-08-06

**Authors:** Tae Wan Kim, So Yeon Koo, Lorenz Studer

**Affiliations:** ^1^The Center for Stem Cell Biology, Developmental Biology Program, Sloan-Kettering Institute for Cancer Research, New York, NY, United States; ^2^Developmental Biology Program, Sloan-Kettering Institute for Cancer Research, New York, NY, United States; ^3^Neuroscience Graduate Program of Weill Cornell Graduate School of Biomedical Sciences, Weill Cornell Medicine, New York, NY, United States

**Keywords:** pluripotent stem cells, Parkinson’s disease, neural transplantation, directed differentiation, dopamine neuron, midbrain development, regenerative medicine

## Abstract

In Parkinson’s disease (PD), there are currently no effective therapies to prevent or slow down disease progression. Cell replacement therapy using human pluripotent stem cell (hPSC)-derived dopamine neurons holds considerable promise. It presents a novel, regenerative strategy, building on the extensive history of fetal tissue grafts and capturing the potential of hPSCs to serve as a scalable and standardized cell source. Progress in establishing protocols for the direct differentiation to midbrain dopamine (mDA) neurons from hPSC have catalyzed the development of cell-based therapies for PD. Consequently, several groups have derived clinical-grade mDA neuron precursors under clinical good manufacture practice condition, which are progressing toward clinical testing in PD patients. Here we will review the current status of the field, discuss the remaining key challenges, and highlight future areas for further improvements of hPSC-based technologies in the clinical translation to PD.

## Introduction

A key promise of human pluripotent stem cells (hPSCs), both human embryonic stem cells (hESCs) and induced pluripotent stem cells (hiPSCs), is their ability to access unlimited number of specialized cell types for application in cell-based therapies for neurodegenerative diseases such as Parkinson’s disease (PD; Fox et al., 2014; Tabar and Studer, 2014; Blau and Daley, 2019). The characteristic, patho-physiological feature of PD is the specific loss of midbrain dopamine (mDA) neurons in the substantia nigra (SN; Mingote et al., 2015), leading to motor symptoms such as bradykinesia, tremor, and rigidity (Lees et al., 2009). Drug treatments, such as L-dopa, for restoring the dopamine deficiency is widely used to relieve disease symptoms, but long-term use shows decreased efficacy and can trigger debilitating side effects including motor complications as well as psychiatric problems (Poewe et al., 2017).

Given the quite selective mDA neuron loss in PD, cell replacement may represent an attractive therapeutic strategy. There has been experience using various sources of cells for transplantation including adrenal medullary tissue and more importantly, human fetal midbrain tissue, starting with human clinical trials more than 30 years ago (Backlund et al., 1985; Goetz et al., 1989; Lindvall et al., 1990; Freed et al., 1992; Widner et al., 1992). More than 300 patients were transplanted using human fetal tissue world-wide, and despite variable results overall, some patients showed remarkable recovery obviating the need for any L-dopa treatment (Kefalopoulou et al., 2014), and robust graft survival has been demonstrated histologically up to 24 years after transplantation (Li et al., 2016). However, the use of human fetal tissue has several limitations such as transplantation of a heterogenous cell population, non-standardized tissue-processing, ethical issues associated with the routine use of fetal tissue, and importantly, the limited availability of suitable tissues. Therefore, alternative cell sources are urgently needed. The use of hPSCs is particularly attractive due to their ability to yield defined lineages such as mDA neurons at scale. Furthermore, hPSCs may provide a more practical and ethically acceptable cell source (Steinbeck and Studer, 2015; Barker et al., 2017; Parmar et al., 2020). The initial challenge in the use of hPSCs was the ability to direct their broad potential toward the restricted production of mDA neuronal lineages. Over the years, several groups have developed suitable protocols for mDA neuron production, and in ongoing or completed developments those protocols have been adapted to generate authentic and functional mDA neurons or precursors under clinical good manufacture practice (cGMP) grade conditions suitable for early stage human trials. Here, we discuss the tremendous progress made over the last decade, review the current bottlenecks, and provide a perspective for the future of developing cell replacement strategies in PD.

## Brief History on mDA Neuron Protocol Development

The initial approach to generate mDA neurons from hPSCs was based on adapting protocols from mouse ESC, which generate the neuronal-rosette like intermediates by co-culturing with feeder such as MS5 and PA6 and then further differentiate mDA neurons (Kawasaki et al., 2000; Perrier et al., 2004; Sonntag et al., 2007). While the rosette-based protocols could yield dopamine neurons that express TH, the rate-limiting enzyme for dopamine production, and showed dopamine release *in vitro*, those cells unlikely represented the correct cell type of origin as they barely expressed floorplate markers, such as FOXA2 and LMX1A. Importantly, rosette-derived dopamine (DA) neuron protocols displayed a considerable risk of neural overgrowth (Brederlau et al., 2006; Roy et al., 2006; Elkabetz et al., 2008), and resulted in only limited *in vivo* DA neuron survival and function (Perrier et al., 2004; Park et al., 2005).

**FIGURE 1:**
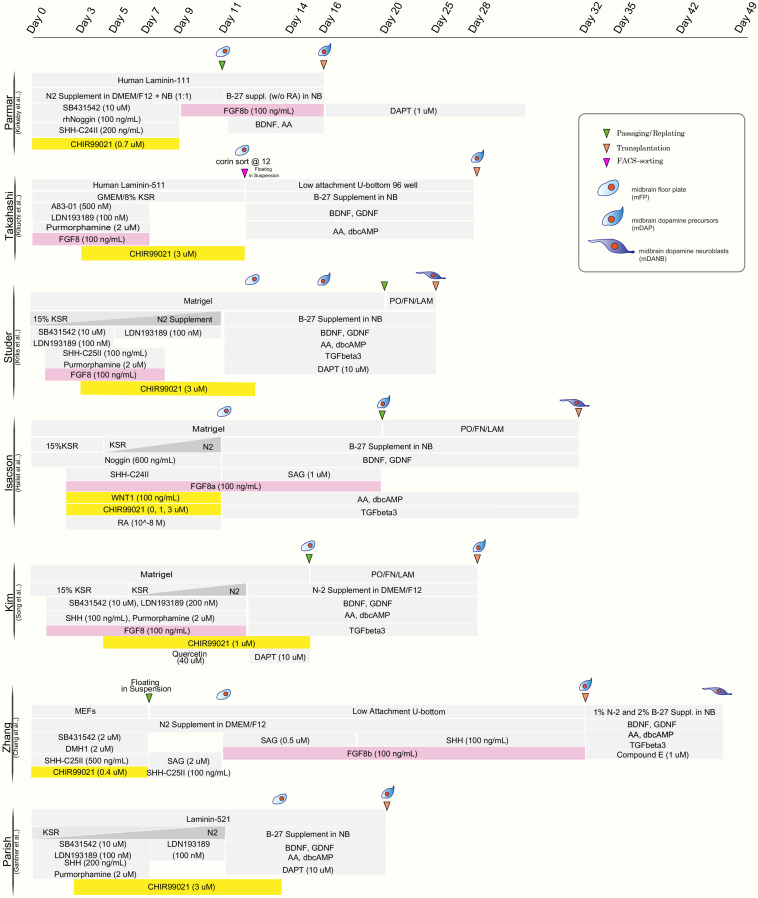
Comparison of published differentiation protocols for dopamine neuron derivation from human pluripotent stem cells. While all protocols use comparable strategies for neural induction (dual-SMAD inhibition) and for midbrain floor plate induction (activation of SHH pathway and WNT pathway), there are differences in the use of FGF8 (highlighted in pink) and the timing and concentration of the WNT activating compounds (typically CHIR99021; highlighted in yellow).

The realization that mDA neurons originate from the midbrain floor-plate (Ono et al., 2007; Bonilla et al., 2008) and the development of improved strategies to drive neural differentiation from hPSCs ignited the development of a new class of protocols. Those protocols used dual SMAD inhibition (inhibition of BMP and TGFβ signaling) for neural induction (Chambers et al., 2009) together with patterning factors activating SHH (Sonic hedgehog), WNT, and FGF8b signaling (Kriks et al., 2011; Figure 1). The resulting floor-plate derived mDA cells showed the biochemical and electrophysiological properties of mDA neurons *in vitro* and resulted in more robust survival and function *in vivo* while reducing the risk of neural overgrowth or teratoma formation (Kriks et al., 2011; Table 1). Since those initial studies, several groups have developed independent mDA neuron differentiation paradigms (Kirkeby et al., 2012, 2017; Sundberg et al., 2013; Doi et al., 2014; Hallett et al., 2014; Steinbeck et al., 2015; Chen et al., 2016; Niclis et al., 2017; Gantner et al., 2020; Song et al., 2020) but all of those are based on the specification of mDA neurons via a floor-plate intermediate (Figure 1).

**TABLE 1:** Comparison of dopamine neuron transplantation paradigm in preclinical studies.

Group	Cell Source	DIV at the time of injection	Cell Dosage for graft	Animal PD Model (Lesioning Method)	Trans-plant Location	Cell Quality Control	Sorting Strategy (N/Y)	mDA Subtypes	Other neuronal types/proliferating cells	References
Parmar	hESC (H9)	Day 16	150,000–300,000 (Striatum)	Rat (6-OHDA MFB)	Striatum	D14 mRNA expression	N	*Intrastriatal grafting* : 80% TH+GIRK2+; 10% TH+CALB+ among total TH+ (Grealish et al., 2014)	PCNA+ (Quantification data N/A) (Kirkeby et al., 2017)	Grealish et al., 2014; Kirkeby et al., 2017
			50,000–75,000 (SNpc)	Rat (6-OHDA MFB)	SN			*Intranigral grafting*: 60% TH+GIRK2+; 20% TH+CALB+ among total TH+ (Grealish et al., 2014)		
Takahashi	hiPSC (healthy vs. PD)	Day 28	400,000	Rat (6-OHDA MFB)	Striatum	80% FOXA2+LMX1A+ (D12); 80% FOXA2+ (D28); 40% TH+ (D42) (Doi et al., 2014)	Y (Corin @ D12)	TH+GIRK2+ (Quantification data N/A) (Kikuchi et al., 2017)	0.06 – 0.07% Ki67+ GABA+, GFAP+ <1% 1.2+/-0.8% 5-HT+ (Doi et al., 2014)	Kikuchi et al., 2017; Doi et al., 2014
			4.8M	Monkey (MPTP IV injection)						
Studer	hESC (H9)	Day 25	150,000–200,000	Mouse (6-OHDA Striatum)	Striatum	FOXA2+LMX1A+>60% (D11); 95% FOXA2+LMX1A+ (D25) 40% FOXA2+NURR1+ (D25); 80% TH+ (D50) 80% FOXA2+ (D50) (Kriks et al., 2011)	N	TH+GIRK2+ and TH+CALB+ (Quantification data N/A) (Kriks et al., 2011)	5-HT (but hNCAM negative); GABA+ <1% (Kriks et al., 2011)	Kriks et al., 2011; Steinbeck et al., 2015
			250,000	Rat (6-OHDA MFB)						
			7.5 M	Monkey (MPTP Carotid followed by IV)						
Isacson	Autologous iPSC	Day 30 OR Day 49	10M–40M	Monkey (MPTP IV administration)	Striatum	21-23% beta-tubulin+ (D28) 9% TH+ (D28) 1-2% TH+FOXA2+ (D28) (Hallett et al., 2015)	N	FOXA2+TH+GIRK2+ (Quantification data N/A) (Sundberg et al., 2013)	No Ki67+ (Hallett et al., 2015)	Hallett et al., 2015; Sundberg et al., 2013
						51% beta-tubulin+ (D47) 7% TH+ (D47) 2% TH+FOXA2+ (D47) (Hallett et al., 2015)				
Kim	hiPSC	Day 28	100,000–300,000	Rat (6-OHDA MFB)	Striatum	FOXA2 +LMX1A +>80% (D28) 40% NURR1+ (D28) 20% TH+ (D28) (Song et al., 2020)	N	79.29+/− 4.88% TH+GIRK2+ among total TH+; TH+ALDH1A1+GIRK2+; TH+ALDH1A1+SOX6+; TH+ALDH1A1+CALB+ (Quantification data N/A) (Song et al., 2020)	5.9% Ki67+ 1.2% SOX1+Ki67+ 0.15% SOX6+PAX6+ No GABA+ or 5-HT+ (Song et al., 2020)	Song et al., 2020
Zhang	hESC (H9)	Day 32	200,000	Rat (6-OHDA SN)	Striatum	EN1 +OTX2+ >80% (D18) FOXA2 +LMX1A +>80% (D18) TH+>60% (D42) 90% FOXA2+EN1+ (D42) (Chen et al., 2016)	N	TH+ALDH1A1+: 61.4+/− 4.8%; TH+GIRK2+: 56.3+/−6.7%; among total TH+ (Xi et al., 2012)	GABA <1% 3.7+/−0.9% 5-HT+ 3.2+/− 0.1% GFAP+ (Chen et al., 2016)	Chen et al., 2016; Xi et al., 2012
Parish	hESC (H9)	Day 20	50,000	Mouse (6-OHDA SN)	Striatum	80% FOXA2 (D11) 90% FOXA2+ (D25) 30% TH+ (D25) (Gantner et al., 2020)	Y (PITX3 ::GFP) (LMX1A ::GFP)	60% PITX3::GFP+GIRK2+; <5% PITX3 ::GFP+CALB+; 20% PITX3::GFP +GIRK2+CALB+; (Gantner et al., 2020)	502+/−11 5-HT+HNA+ cells (Gantner et al., 2020)	Gantner et al., 2020

*Representative studies are presented across different research groups that are working toward translating dopamine neuron cell therapy in PD. Comparison of key *in vitro* and *in vivo* parameters are presented based on the data taken from the published reports.*

One interesting comparator to assess the potency of hPSC-derived mDA neurons was their preclinical assessment in direct comparison to the efficacy of human fetal tissue grafts showing comparable results in rescuing dopamine deficits in a PD rat model and in achieving proper target-specific neurite-outgrowth (Grealish et al., 2014). In another set of studies, *in vivo* graft function of mDA cells was explored in preclinical PD animal models using more sophisticated technologies such as optogenetics (Steinbeck et al., 2015) and chemogenetic manipulation (Chen et al., 2016). Those studies demonstrated conclusively that the recovery from PD behavior is dependent on neuronal activity and activity-dependent dopamine release from transplanted mDA cells. Finally, differentiation protocols to generate floor-plate-derived mDA neurons demonstrated their robustness and reproducibility by developing mDA cell products under conditions that should be suitable for cGMP manufacturing (Kikuchi et al., 2017; Kirkeby et al., 2017; Figure 1). In an effort to promote the safe transition of hPSC-derived mDA neurons toward clinical testing in PD patients, a global effort, G-Force-PD was initiated in 2015 (Barker et al., 2015). G-Force-PD includes several groups from the United States, Europe, and Japan which all are leading independent efforts to bring hESC or hiPSC-derived mDA neurons to the clinic, but are committed to sharing their ongoing experience, discuss unexpected challenges and possible solutions, and propose guidelines ranging from cell manufacturing to clinical trials design (Barker et al., 2017). One of the groups within G-Force-PD has already started a first in human clinical cell transplantation trial in PD patients using iPSC-derived mDA neurons, with the first patient grafted in October 2018 (Normile, 2018)^1^. Another group has just recently published the feasibility of transplanting one single patient with its own iPSC-derived mDA neurons (Schweitzer et al., 2020).

## What Cell Type-Related Factors Are Critical for Clinical Translation?

### Proper Patterning of mDA Precursors Beyond Establishing Floor-Plate Precursor Identity

Although most protocols have been able to generate floor-plate precursors at high efficiency, various groups have used different timing, duration, and concentration of patterning factors, specially CHIR99021 (CHIR), the most widely used GSK3-inhibitor for triggering canonical WNT activation and recombinant factor FGF8 which is added in some but not other protocols for distinct time periods of differentiation (Figure 1). The precise titration of CHIR is critical for proper rostro-caudal pattering during mDA neuron specification from hPSC. Work by the Parmar lab (Kirkeby et al., 2012) demonstrated that different levels of WNT activation trigger dose-dependent changes in the regional identity of neuronal progenitor cells ranging from telencephalon (low WNT activation) to hindbrain (high WNT activation) fates, with the optimized CHIR concentration for mDA neuron induction defined as (0.7–0.8 μM). Furthermore, single cell RNA sequencing of Lmx1a+ precursors in the mouse suggests that the midbrain floor-plate markers FOXA2 and LMX1A are not specific to just mDA progenitors, but also mark the more anterior, subthalamic nucleus (STN) precursors which go on to give rise to glutamatergic neurons (Kee et al., 2017). Those results are in agreement with the idea that the midbrain-diencephalic floor-plate region is subdivided into two distinct domains by En1 for posterior, midbrain (mDA) identity, and Dbx1 for STN neuron population (Nouri and Awatramani, 2017). Importantly, lowering CHIR exposure (0.4–0.6 μM) resulted in a bias toward Lmx1a+/Pitx2+ positive cells (STN neuron) while the percentage of STN neurons was greatly decreased at higher concentrations of CHIR (0.8–1 μM) with increased efficiencies of mDA neuron differentiation (Kee et al., 2017). Nevertheless, a broad range of concentrations and durations of CHIR treatment have been proposed across for mDA neuron derivation from hPSCs as detailed in Figure 1. There are several possibilities for those discrepancies across protocols. In particular, the basal media composition (KSR versus N2 versus E6 media), coating substrates (Matrigel versus LAM511) and the total lengths of CHIR treatment likely contribute to those differences.

Studies, using bulk-RNA sequencing of mDA neurons at the time of grafting demonstrated a correlation of improved *in vivo* graft outcome, across >30 batches, for mDA neuron populations expressing more caudal floor-plate markers including expression of EN1 (Kirkeby et al., 2017). Furthermore, the same study showed that FGF8b treatment after floor-plate induction could more reliably induce caudal marker expression. These results were consistent with previous work suggesting the need for FGF8 treatment at later time points of differentiation during non-human primate mDA neuron induction (Xi et al., 2012; Figure 1). In contrast, earlier exposure of FGF8b during mDA patterning may not impact the robustness of midbrain marker expression (Kriks et al., 2011). One challenge of protracted FGF8b treatment is the fact that FGF8b is highly expressed in the hindbrain during early development (Liu et al., 2003). Therefore, induction of hindbrain markers, such as HOXA2 and GBX2, can occur following high dose FGF8b treatment (Kirkeby et al., 2017). Thus, the use of FGF8 needs to be very carefully timed and titrated to avoid hindbrain and potentially other FGF8-driven proliferative contaminants. Alternatively, it may be possible to find alternative strategies such as the use of midbrain-specific FGFs (Liu et al., 2003) to substitute for late FGF8b treatment or to develop strategies to selectively enrich for the desired phenotype for translational applications.

Another remaining challenge is to selectively generate populations of specific mDA neuron subtypes including A9 (SN) or A10 (Ventral tegmental area; VTA) mDA neurons. A9 mDA neurons are particularly vulnerable in PD (Damier et al., 1999; Surmeier et al., 2017) and are the main cell type of interest for cell therapy. Thus far, most studies used immunolabeling for two widely used markers, GIRK2 for A9 and CALBINDIN for A10 (Chung et al., 2005) to characterize mDA neurons subtypes *in vitro* or upon transplantation. Several studies reported the presence of ∼60–80% of GIRK2 + TH + neurons and ∼10–20% of CALB1 + TH+ in dorso-striatal grafts after 4.5–6 months post hPSC-mDA neuron transplantation (Table 1). However, those two markers are likely insufficient to segregate A9 versus A10 identity, and they may be particularly less reliable at early, prenatal stages of development. Importantly, neither marker is suitable to prospectively distinguish mDA neuron subtypes at the time of transplantation.

Other subtype selective markers of interest include ALDH1A1, which has been defined as an A9 type marker (Chung et al., 2011; Xi et al., 2012; Song et al., 2020). Both ALDH1A1 and the transcription factor SOX6 have been reported as specific molecular determinants expressed at the mDA neuron progenitor stage, marking precursors that later develop into ventro-lateral A9-type SN neurons (Panman et al., 2014; Blaess and Ang, 2015). Accordingly, several studies strived to induce the expression of ALDH1A1 and SOX6 during hPSC-differentiation. R-Spondin 2 (Gyllborg et al., 2018) and BMP5/7 (Jovanovic et al., 2018) were reported as candidate inducers of ALDH1A1 and SOX6 respectively *in vitro*. R-Spondin 2 treatment during floor-plate patterning increased ALDH1A1 expression by 5-fold compared to control by gene expression, but it remained unclear how those gene expression changes correspond to protein expression. Therefore, it will be important to determine the total numbers of ALDH1A1+/TH+ neurons that can be achieved under those conditions and the level of their functionality *in vivo* (Gyllborg et al., 2018). Similarly, despite promising data in the mouse, where BMP signaling mutants showed reduced numbers of A9 neurons postnatally, BMP5/7 exposure appeared to have minimal effects on enriching A9 mDA neurons *in vitro* and without clear information on the percentages of SOX6 + TH + neurons that can be obtained under those conditions (Jovanovic et al., 2018).

Despite many years of work using GIRK2, SOX6, and ALDH1A1 as individual A9 markers to characterize mDA neurons, the growing number of scRNAseq studies on mDA neuron subtypes (Poulin et al., 2014; La Manno et al., 2016; Tiklova et al., 2019) indicate that it is essential to multiplex markers for reliable subtype identification. For example, ALDH1A1 and SOX6 single positive cells can be found both in VTA and SN domains, whereas co-expression should identify SN neurons at the ventro-lateral location within the midbrain (Poulin et al., 2020). Given the availability of multiple protocols to efficiently derive floor-plate-derived mDA precursors, a key next step is to define the signaling cascades that restrict those precursors into pure mDA neurons (versus other floor-plate-derived neuronal lineages) and further into selective mDA neuron subtypes. Those efforts should go hand in hand with improved strategies to identify markers that capture stage and subtype identity during early developmental stages, stages relevant for cell transplantation.

### Homogenous mDA Cell Population

The application of hPSC derivatives in human patients has progressed slowly due to potential risks associated with the use of pluripotent stem cells, where a few contaminating hPSCs could proliferate and develop into a teratoma or into early stage neuroepithelial tumors (Brederlau et al., 2006; Sonntag et al., 2007; Elkabetz et al., 2008). Furthermore, the transplantation of heterogenous neuronal population may entail the risk of side effects such as graft-induced dyskinesia (Dorsey et al., 2018) which have been attributed to the presence of serotonergic neurons in fetal grafts, as documented in grafted human PD patients by histological analysis and by functional studies using pharmacological and PET based assays (Politis et al., 2010). Without understanding the potential risks and side-effects that could come from “off-target” neuronal or non-neuronal populations, most groups strive to produce highly enriched and defined mDA neuron populations and to eliminate any contaminating cells to assure safety for clinical translation. Several strategies have been proposed to avoid unwanted cells either via enriching floor-plate or later stage mDA precursors using a surface marker (Doi et al., 2014; Samata et al., 2016; Lehnen et al., 2017) or via eliminating remaining undifferentiated hPSCs by exposing cultures during mDA neuron differentiation with natural compound, quercetin (Song et al., 2020). Those studies reported that purified mDA progenitors resulted in more homogenous graft size and mDA neuron density as well as in improved recovering motor dysfunctions in PD animal models than non-sorted cells without evidence of any tumor formation (Doi et al., 2014; Samata et al., 2016; Lehnen et al., 2017). However, while some markers such as Corin can enrich for floor-plate precursors, and NCAM or ALCAM (Bye et al., 2015) can enrich for certain neural cells, none of the currently available markers seems to be truly specific for mDA neuron lineage. Accordingly, there is currently no consensus on any strategy to enrich for mDA neurons reliably and new technologies will be needed for successful clinical translation. One strategy that appears capable of generating near homogenous populations of mDA neurons is to use genetic reporter PSC lines that mark either mDA progenitors or their post-mitotic progeny. We have recently reported that NURR1 (NR4A2) can serve as a reliable marker of early post-mitotic mDA neurons under floor-plate differentiation conditions. An NURR1:H2B-GFP reporter line allowed enrichment of mDA neurons *in vitro* that yielded nearly pure populations based on mDA markers such as FOXA2, LMX1A, NURR1 and TH including by using flow based and sc-RT-qPCR analyses (Riessland et al., 2019). While genetic engineering and FACS-based purification of neurons presents major challenges of human translation, it may be possible to adapt this approach for MACS-based or genetic selection strategies more suitable or translation. In either case, the use of genetic reporters is a powerful tool to define the appropriate stage of mDA neuron development for transplantation (see below). Similarly, once we better define early markers that can segregate mDA neurons, we can also apply those and other novel markers to selectively isolate A9-versus A10 type mDA neurons *in vitro*.

### Stage of mDA Cell for Grafting

Over the last decades, fetal tissue transplantation studies have provided tremendous insights how to reconstruct the damaged brain of a PD patient. One such aspect was the discovery of the optimal window for isolating mesencephalic cells from fetal tissue. Human mDA neurogenesis occurs during a narrow window of early CNS development at week 6–8.5 p.c. (Olson et al., 1973; Freeman et al., 1991). Interestingly, human fetal grafts derived from tissue later than 9 weeks showed reduced survival, particularly when injected as cell suspension, and did not reliably yield functional mDA neurons *in vivo* (Freeman et al., 1995; Almqvist et al., 1996; Olanow et al., 1996). In contrast, tissue derived from fetuses at 5.5–8 weeks p.c. has yielded the best results in pre-clinical and clinical results with robust survival of mDA neuron rich grafts. One additional important point regarding the age of the donor may be the differential yield in production of mDA neuronal subtypes. Rodent studies have implicated that the younger E10 tissue gave rise to ∼75% GIRK2 + mDA neurons in intrastriatal grafts whereas older E12 tissue contributed 60% of GIRK2 + mDA neurons possibly due to staggered birth timing of A9 versus A10 mDA neurons (Gates et al., 2006; Bye et al., 2012).

Given the extensive push to develop floor-plate derived mDA neurons from hPSCs, it is essential to address questions about optimal stage of *in vitro*-derived cells for transplantation. From a simple, regenerative medicine perspective, one could argue that it makes the most sense to replace what is lost in PD, which are mature mDA neurons. However, as learned from the fetal tissue experience, mature mDA neurons may not survive upon transplantation into the adult brain, and thereby do not represent a viable option.

To systematically determine the optimal stage for transplantation, we previously reported in mouse ESCs on a side-by-side comparison of the engraftment potential of Hes5:GFP + progenitors, vs Nurr1 + neuroblasts vs Pitx3 + mDA neurons *in vivo* (Ganat et al., 2012). Pitx3 + mDA neurons survived only poorly resulting in smaller grafts with lower numbers of total mDA neurons and with worse behavioral data in drug-induced rotation tests as compared to both Hes5 + or Nurr1 + grafts. Our results indicated that Nurr1 may capture a suitable stage for transplantation while Pitx3 marks mDA neurons that are too mature for efficient *in vivo* engraftment. Interestingly, the Pitx3 finding was recently confirmed in an independent study using human ESC genetic reporter lines for PITX3 and for LMX1A (de Luzy et al., 2019). While PITX3 + mDA neuron showed very poor survival, LMX1A + cells at the floor-plate precursor stage showed efficient engraftment, albeit the resulting grafts (similar to Corin + grafts) were far from representing pure mDA neuron grafts (Gantner et al., 2020).

The identification of Nurr1 + stage a suitable for grafting has been translated into human PSC-based differentiation studies where mDA neurons were isolated at day 25 of differentiation, the stage of high NURR1 expression when most cells have started to exit cell cycle. Such early post-mitotic neurons appear to produce rich mDA neuron grafts and have resulted in successful rescue of complex motor abnormalities in animal PD models including mice, rat, and monkey (Kriks et al., 2011; Ganat et al., 2012; Steinbeck et al., 2015). Other teams such as the group by Isacson and Kim used similar strategy to transplant their cells after expanding mDA progenitors for a certain period (Song et al., 2020). Jun Takahashi’s group transplanted later, day 28 mDA “progenitors,” which are differentiated further starting from floor-plate progenitor stage sorted by CORIN at day 12 (Doi et al., 2014; Kikuchi et al., 2017). In contrast, the Parmar team demonstrated that relatively early stage, day 16 mDA progenitors, just beyond floor-plate precursor stage are also suitable for intracerebral transplantations (Kirkeby et al., 2012, 2017; Grealish et al., 2014, 2015; Cardoso et al., 2018; Adler et al., 2019). Despite the relatively earlier time point for transplantation, the protocol strives to transition the progenitors toward a “neural” fate via concurrent treatment of FGF8b (Kirkeby et al., 2017; Nolbrant et al., 2017), BDNF, and ascorbic acid (AA) (Yan et al., 2001) for about a week to achieve progenitor cell expansion and *in vitro* differentiation toward mDA neuron stage. In conclusion, the transplantation of the cells starting from late midbrain floor-plate to an mDA neuroblast stage or early mDA neuron may be all suitable for robust survival and function, but additional side-by-side transplantation studies using human PSCs will be required to fully explore this point, particularly for the mDA stage-dependent extent of DA fiber outgrowth and synaptic integration.

A parallel strategy to further improve graft composition is the generation of extensive molecular data on hPSC-derived mDA cells prior to transplantation and correlating those data with the subsequent performance of grafted cells (Kirkeby et al., 2017; Tiklova et al., 2020). This strategy should allow the iterative optimization of graft composition to fine-tune both subtype identity and stage of the grafted cells for optimal functional results. Such technology can also be further combined with the use of prospective lineage tracking and barcoding methods to link cell identity to graft outcome.

### Location of the Cell Injected

Cell transplantation in PD has mainly focused on ectopic placement of cells within the striatal target region, far away from the site of degeneration in the SN. The rationale for this approach arose from the concern that the extent of the mDA axonal outgrowth from the graft may not suffice to efficiently innervate the human caudate or putamen. Additionally, in some of the studies, DA neurons did not survive as well when grafted in the midbrain compared to striatum (Thompson and Bjorklund, 2012). Furthermore, grafting studies over the last decade have demonstrated that intra-striatal grafting of fetal tissue is sufficient for restoring striatal dopamine release and inducing recovery from PD-relevant symptoms at least a subset of patients (Kefalopoulou et al., 2014). Ectopic transplantation, however, raises the concern that grafted cells may lack major afferent inputs of endogenous mDA neurons in SN, inputs known to play important roles in phasic regulation of nigrostriatal neuron activity. This lack of afferent control may restrict the ability of the cells to improve more complex motor behaviors (Winkler et al., 2000; Björklund et al., 2003). Furthermore, there is increasing evidence that DA released from dendrites locally in the SN may have important physiological functions distinct from striatal DA release (Cheramy et al., 1981). Therefore, several groups have pursued a long-term goal of orthotopic transplantation using hPSCs-derived mDA cells into the SN.

The Parmar group demonstrated that mDA progenitors grafted into the murine SN can extend long axons over 10 mm from the graft core innervating various rat brain structures including caudate-putamen, nucleus accumbens, and amygdala (Grealish et al., 2015). Using pseudo rabies-virus-based retrograde, monosynaptic tracing technology (Grealish et al., 2015; Cardoso et al., 2018), they demonstrated the establishment of afferent inputs on the grafted cells from the host starting by 6 weeks post transplantation (Grealish et al., 2015). Those inputs appear largely dependent on graft placement (Grealish et al., 2015; Cardoso et al., 2018). However, it remains unclear to what extent differences in afferent synaptic inputs, such as exaggerated thalamic but reduced hypothalamic and raphe inputs, affect the functional behavior of striatal versus nigral placed mDA cell grafts (Adler et al., 2019).

Furthermore, the behavioral readouts in intranigral grafting studies were typically limited to drug-induced rotation tests, which are not dependent on afferent input of the transplant (Winkler et al., 2000). Therefore, it would be interesting to test whether intranigral grafting can trigger rescue of more complex motor behaviors and whether those are dependent on afferent input or possibly on nigral DA release. Additionally, the human brain is much larger than rodent or primate brains, and thus homotopic grafting may be more challenging to achieve meaningful projections from the SN to appropriate regions within the striatum such as the post-commissural putamen. In human fetal tissue grafting studies, SN injections have been attempted but only in combination with striatal injections (Mendez et al., 2005), which makes it difficult to assess the relative contribution of each site to any clinical parameters. However, the technology to deliver hPSC-derived mDA progenitors in the SN will likely improve, and optimization of graft composition, the addition of growth promoting factors or even exercise of the host brain may contribute to achieve reliable restoration of the nigro-striatal circuit (Torikoshi et al., 2020).

### mDA Cell Dosage for Transplantation

Typically, 200,000–420,000 dopamine neurons reside in human midbrain, and it is estimated that 50% loss of those DA neurons leads to the PD symptom (Brichta and Greengard, 2014). According to preclinical studies using fetal tissue or hPSC-derived mDA cell, 1200–2400 surviving TH + neurons in rat, 13,000 in primate, 40,000–80,000 in the human brain may be required to achieve a meaningful therapeutic effect (Hallett et al., 2015; Harris et al., 2020). The current bottleneck in delivering cells to the brain is that typically less than 10% of grafted mDA neurons survive following transplantation (Brundin et al., 2000; Thompson and Bjorklund, 2012; Hallett et al., 2014). Multiple factor may contribute to poor mDA neuron survival including mechanical trauma, growth factor deprivation, initial lack of vascularization, hypoxia and free radical production, or excessive extracellular concentrations of excitatory amino acids in the host brain (Brundin et al., 2000).

The Takahashi group embarked on the first human clinical trial by injecting 2.4 million iPSC-derived mDA cells in 2018. The Kim group injected 4 million iPSC-derived mDA precursors in one patient (Schweitzer et al., 2020). Upcoming human clinical trials in New York and Europe propose two different starting doses – high and low – for their proposed early stage (Phase I/IIa) clinical trials to assess the feasibility and safety of various dosages (Barker et al., 2017). Several studies have been proposed to enhance fetal or PSC-derived mDA neuron grafts such as by promoting their survival or function. Examples include treatment with pifithrin-alpha, increase of polysialic acid levels (Chou et al., 2011; Battista et al., 2014), or delivery of neurotrophic factors such as glial cell line-derived neurotrophic factor (GDNF) which may provide benefits to facilitate graft integration and functional recovery (Rosenblad et al., 1996; Redmond et al., 2009; Gantner et al., 2020).

## Next Generation of mDA Cell Products

### Immuno-Compatible mDA Neuron

Past clinical fetal and hPSC-based studies have mostly focused on the use of allogenic cell sources for transplantation. Given the relatively mild reaction to allogenic grafts in the CNS, the use of only transient immunosuppression offers long-term graft survival for >20 years (Li et al., 2016). In fact, some groups have performed fetal transplantation without any immunosuppression and showed long-term graft survival, albeit possibly with reduced mDA neuron numbers. Studies in the mouse (Tabar et al., 2008) and in primates (Morizane et al., 2017) showed concordant results suggesting that allografting results in mDA neuron survival but at lower rates, particularly if there is a considerable mismatch between host and graft. While those differences seem to disappear in the presence of transient immunosuppression (Morizane et al., 2017) grafting matched cells back into non-human primate hosts has been demonstrated by several groups (Hallett et al., 2015; Kikuchi et al., 2017) and most recently in one human individual (Schweitzer et al., 2020).

However, an isogenic mDA neuron approach raises the issue whether those patient-specific neurons may have a genetic predisposition that will make them more prone to succumb to disease after transplantation. In addition, such an approach is labor intensive and costly which will complicate future clinical implementation. In particular, it will be more difficult to establish extensive safety data for the cells in each individual patient and thereby increase clinical risk. Instead, transplantation of human leucocyte antigen (HLA)-matched iPSC-derived mDA cells has been proposed to minimize the risk of allograft rejection (Taylor et al., 2012). There are several ongoing efforts world-wide to establish HLA-homozygous iPSC lines that can match large proportions of the overall population. In fact, the first human clinical trial in Japan made use of one such HLA-homozygous lines for transplantation in PD (Normile, 2018), albeit still using transient immunosuppression.

Alternatively, there is considerable excitement in generating universal hPSC lines, which may provide immune tolerance without any immunosuppression. Using such an approach, theoretically, mDA neurons from a single universal hPSC line could be administrated to any PD patient worldwide. Proof-of-concept in establishing universal hPSC has achieved either by the combination of B2M gene knock-out with HLA-E overexpression (Gornalusse et al., 2017) or by knock-out of major histocompatibility complex (MHC) class I/II with CD47 (do not eat me signal) overexpression (Deuse et al., 2019). Given the potential of such engineered, universal cells to escape immune surveillance, implanting these cells should be pursued very carefully to avoid concerns regarding safety. In fact, those strategies are typically combined with integrating a drug-induced suicide switch in the cells as a failsafe (Gornalusse et al., 2017; Deuse et al., 2019).

### Pathology-Resistant mDA Neuron

Lewy body formation is well known as one of the neuro-pathological hallmarks in PD (Braak et al., 2003), Interestingly, while no disease pathology was observed in PD patients over the first 10 years after transplantation, Lewy bodies have been identified in human fetal tissue grafts starting 11–16 years after the cell therapy with increasing percentages of affected cells by >20 years post grafting. As grafted cells were fetal tissue-derived and unlikely to have a PD predisposition, such Lewy body pathology suggests host-to-graft disease propagation (Kordower et al., 2008; Li et al., 2008). While progressing very slowly, such transmission could ultimately be toxic to transplanted mDA cells and limit the long-term efficacy of the cell therapy. Previous work suggests that both cytoplasmic insoluble and endogenous soluble alpha-synuclein protein is necessary to form the Lewy body and exert toxic effects on mDA neurons (Luk et al., 2009). Therefore, SNCA (alpha-synuclein) knockout (KO) or knock-down hPSC lines (Chen et al., 2019) may be an attractive strategy to yield pathology-resistant mDA neurons. The feasibility of this approach is further supported by the lack of major functional deficits in SNCA KO mice (Abeliovich et al., 2000).

### Pure Substantia Nigra (A9-type) mDA Neuron

Recently, single cell gene profiling has been used to define subtype compositions during mouse and human midbrain development (Poulin et al., 2014; La Manno et al., 2016; Kee et al., 2017; Tiklova et al., 2019). Such technology extended the previous molecular definition of mDA neurons and enabled to further divide SN and VTA region into seven distinct molecular clusters (Poulin et al., 2020). However, to what extent these molecular findings can be translated into hPSC-derived mDA neuron development remains unexplored. This is illustrated when profiling of hPSC-derived mDA neurons in La Manno et al., which seem to recapitulate key stages of *in vivo* ventral midbrain development. However, those cell preparations expressed many poorly defined radial glial and neuroblast markers and differed from the *in vivo* phenotypes in gene expression (La Manno et al., 2016). Additionally, ALDH1A1 was not expressed in any of those PSC-derived mDA cells. This result may be due to the *in vitro* culture environment that does not fully support mDA neuron development or lack of proper induction of certain subtype-specific genes. In either case, those results indicate that there is considerable room for further improvements in mDA neuron derivation and maturation strategies.

The developmental ontogeny of distinct mDA neuron subtypes remains a long-standing question in the field. Now with rapidly evolving high throughput sequencing technology and lineage tracking tools, it becomes possible to revisit questions about ontogeny and to monitor the developmental trajectory at a single cell resolution from the acquisition of mDA precursor stage both *in vitro* to a fully functional mDA neurons many months after transplantation *in vivo*. Such data should enable a next generation of mDA neuron protocols to reap the full benefit of this approach for human clinical transplantation studies of the future.

## Conclusion

Given the extensive history of cell replacement therapy using human fetal tissue and the rapid recent advances in human stem cell technology, there is considerable current activity around establishing and translating clinical-grade protocols into early stage trials in PD patients. However, despite the progress made thus far and results from the first clinical studies emerging soon, the scientific effort to develop improved grafting strategies should not stop here. It remains essential to carefully consider and address remaining bottlenecks in the field as reviewed in this article. Next generation of mDA cell products should address those remaining barriers on the road to making cell replacement possibly a routine therapeutic strategy that could become available to PD patients world-wide. Only if basic and translational efforts continue hand in hand, we will be able to capture the full potential of this approach in PD and pave the way for other regenerative approaches in brain repair.

## Author Contributions

All authors contributed to the writing, reviewing, and editing the manuscript, and to the preparation of figures and tables.

## Conflict of Interest

LS is a scientific founder and paid consultant of BlueRock Therapeutics and an inventor on patents related to the differentiation of dopamine neurons from pluripotent stem cells. The remaining authors declare that the research was conducted in the absence of any commercial or financial relationships that could be construed as a potential conflict of interest.

## Footnotes

1 https://www.sciencemag.org/news/2018/07/first-its-kind-clinical-trial-will-use-reprogramed-adult-stem-cells-treat-parkinson-s
